# Vasodilation through levodopa for Parkinson's disease may require high left ventricular assist device flow

**DOI:** 10.1111/jocs.14012

**Published:** 2019-03-08

**Authors:** Corstiaan A. den Uil, Edith C. H. Friesema, Alina A. Constantinescu

**Affiliations:** ^1^ Department of Cardiology, Mechanical Circulatory Support and Cardiac Transplantation University Medical Center, Thoraxcenter, Erasmus University Medical Center Rotterdam the Netherlands; ^2^ Department of Intensive Care Medicine University Medical Center, Thoraxcenter, Erasmus University Medical Center Rotterdam the Netherlands; ^3^ Department of Internal Medicine University Medical Center, Section of Pharmacology, Vascular and Metabolic Diseases, Erasmus University Medical Center Rotterdam the Netherlands

**Keywords:** cardiovascular pathology, perfusion

## Abstract

We report implantation of a left ventricular assist device (LVAD) in a patient with Parkinson's disease. Postoperative fluid overload together with insufficient LVAD output in the setting of vasodilation through levodopa likely caused renal hypoperfusion and acute kidney injury. A patient like ours, therefore, requires the highest possible increase of HM3 RPM and LVAD flow early after surgery.

A 52‐year‐old male was admitted with INTERMACS III nonischemic advanced heart failure. He had stable Parkinson's disease treated with oral levodopa/carbidopa (200/50 mg SR and 150/37.5 mg 5 t.d.). His left ventricle was severely dilated end‐diastolic diameter (EDD 94 mm). Filling pressures were high (central venous pressure [CVP] 17 mm Hg, pulmonary capillary wedge pressure [PCWP] 41 mm Hg, transpulmonary gradient 7 mm Hg). Fick calculated cardiac output (CO) was 3.8 L/minute.

He underwent uncomplicated HeartMate 3 implantation. The postoperative course was marked by fatigue, but he was discharged to the ward on day 5. He then developed progressive dyspnea and renal failure. Two weeks after surgery, he was readmitted to the ICU with pulmonary edema and anuric KDIGO stage 3 acute kidney injury (AKI). LVAD parameters were normal (5200 RPM, flow 4.0 L/min, power 3.8, pulse index 3.5). The NT‐pro‐BNP level was 7319 pmol/L. The patient was intubated. Right heart catheterization revealed: CO 5.1 L/min/m^2^, systemic vascular resistance (SVR) 722 dynes/sec/cm^5^, CVP 14 mm Hg, PCWP 23 mm Hg, and SvO_2_ 0.61. Transoesophageal echocardiography demonstrated a dilated LV, rightward bulging of the septum, and frequent aortic valve opening. Rotations were increased to 6200 RPM to produce an LVAD flow of 5.5 L/min, CO 8.5 L/min, and SvO_2_ 0.72. This change in settings led to 1:2 aortic valve opening, an immediate increase of diuresis, resolution of pulmonary edema, and full recovery of renal function.

What caused his normal‐output “heart” failure? Blood cultures were negative. We did not find endocrine pathology. Interrogation of the device represented a normal function. Chest computed tomography demonstrated normal positions of both LVAD and vascular prosthesis. We hypothesized that levodopa may exert systemic vasodilatory effects for which a higher output could be necessary (Figure 1).^1^ To investigate this hypothesis, catecholamine levels were measured according to the available standard extraction and derivation procedure using high‐performance liquid chromatography with fluorometric detection.^2^ After informed consent, blood was collected via an intravenous line, every 4 hours, during 48 hours, after 15 minutes of patient rest. No vasoactive drugs were administered. Although the method that we used could not reliably separate levodopa and dopamine levels, all measured “dopamine” levels were highly elevated (median 635, range 183‐1080 pg/mL; normal < 60 pg/mL). One month later, predischarge, the same results were obtained (457 [175‐1564] pg/mL). The patient's 6‐minute walk distance at discharge was 362 m (normal: > 380 m).

**Figure 1:**
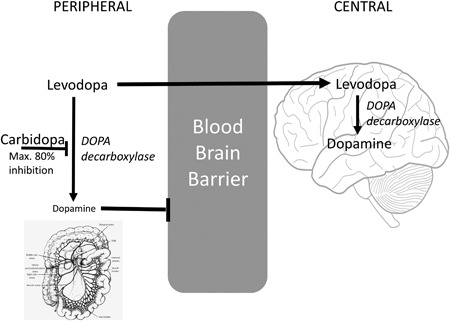
Levodopa is converted to dopamine via the action of a naturally occurring enzyme called DOPA decarboxylase. This occurs both in the peripheral circulation and in the central nervous system after levodopa has crossed the blood‐brainbarrier. Activation of central dopamine receptors improves the symptoms of Parkinson's disease; however, activation of peripheral dopamine receptors causes splanchnic dilation and nausea. For this reason, levodopa is usually administered in combination with a DOPA decarboxylase inhibitor, in this case, carbidopa, which is polar and charged at physiologic pH and cannot cross the blood brain barrier. However, it prevents the peripheral conversion of levodopa to dopamine and thereby reduces the unwanted peripheral side effects of levodopa and increases the quantity of levodopa in the bloodstream that is available to enter the brain. Maximal DOPA decarboxylase inhibition by carbidopa in man is approximately 80%^4^

## CONCLUSION

This is the first report of implantation of an LVAD in a patient with Parkinson's disease on levodopa. Postsynaptic DA1 receptor stimulation by dopamine from incomplete DOPA decarboxylase inhibition, although not definitely proven due to the indistinguishable assay that we used, produces splanchnic vasodilation. These effects may be aggravated by central inhibition of the sympathetic nervous system.^3^ Postoperative fluid overload together with insufficient LVAD output in the setting of vasodilation likely caused renal hypoperfusion and AKI. A patient like ours therefore requires the highest possible increase of HM3 RPM and LVAD flow early after surgery.

## AUTHOR CONTRIBUTIONS

CAU conceptualized, designed, drafted, and approved the article. ECF and AAC critically revised and approved the article, and ECF collected data.

## References

[1] Goldberg LI , Whitsett TL . Cardiovascular effects of levodopa. JAMA. 1971;218(13):1921‐1923. 5171068

[2] van der Hoorn FA , Boomsma F , Man in 't Veld AJ , Schalekamp MA . Determination of catecholamines in human plasma by high‐performance liquid chromatography: comparison between a new method with fluorescence detection and an established method with electrochemical detection. J Chromatogr. 1989;487(1):17‐28. 2715261 10.1016/s0378-4347(00)83003-0

[3] Murphy MB , Elliott WJ . Dopamine and dopamine receptor agonists in cardiovascular therapy. Crit Care Med. 1990;18(1 Pt 2):S14‐S18. 1967161

[4] Robertson DR , Wood ND , Everest H , et al. The effect of age on the pharmacokinetics of levodopa administered alone and in the presence of carbidopa. Br J Clin Pharmacol. 1989;28(1):61‐69. 2775615 10.1111/j.1365-2125.1989.tb03506.x PMC1379971

